# The impact of long-term care on primary care doctor consultations for people over 75 years

**DOI:** 10.1007/s10198-018-0999-6

**Published:** 2018-09-05

**Authors:** Julien Forder, Katerina Gousia, Eirini-Christina Saloniki

**Affiliations:** 1 Personal Social Services Research Unit, University of Kent, Canterbury, Kent CT2 7NX UK; 2 Centre for Health Services Studies, University of Kent, Canterbury, Kent CT2 7NX UK

**Keywords:** Substitution, Social care, Primary care, Older people, I11, I12, I19

## Abstract

Many countries are adopting policies to create greater coordination and integration between acute and long-term care services. This policy is predicated on the assumption that these service areas have interdependent outcomes for patients. In this paper, we study the interdependencies between the long-term (home care) services and consultations with a primary care doctor, as used by people over 75 years. Starting with a model of individual’s demand for doctor consultations, given supply, we formalize the hypothesis that exogenous increases to home care supply will reduce the number of consultations where these services are technical substitutes. Furthermore, greater coordination of public service planning and use of pooled budgets could lead to better outcomes because planners can account for these externalities. We test our main hypothesis using data from the British Household Panel Study for 1991–2009. To address potential concerns about endogeneity, we use a set of instrumental variables for home care motivated by institutional features of the social care system. We find that there is a statistically significant substitution effect between home care and doctor visits, which is robust across a range of specifications. This result has implications for policies that consider increased coordination between health care and social care systems.

**Electronic supplementary material:**

The online version of this article (doi:10.1007/s10198-018-0999-6) contains supplementary material, which is available to authorized users.

## Introduction

In England and in a number of other countries, long-term care (LTC) systems are organised and funded separately from (acute) health services [1, 2]. Yet, the ageing of populations and changes in the complexity of health and care needs are calling these arrangements into question. For England, the Department of Health has projected that by 2026 there will be more than 6 million people aged over 75 years and by 2018 3 million people will have three or more long-term conditions, whether physical, mental or both [3]. Similar ageing patterns are predicted in many other countries. Older people are heavy users of health and LTC services, accounting for the largest part of the total health and long-term (social) care spending in England [3]. In this context, concern has been raised about the problems of separate systems, not least the duplication of services, delays in the provision of care, failure to prevent onset of needs and patient dissatisfaction [4]. More generally, a range of barriers (administrative, financial and cultural) have been identified that limit the coordination of the two sectors [5–7].

In recent years, a range of normative policy arguments have been made that better integration and coordination of care can address these challenges and lead to better outcomes [4]. Some permissive policies have also been put in place, although in practice, there appears to have been little actual coordination activity [8]. More recently, a ‘shared commitment’ to integrated care and support was agreed [4] following a White Paper, *Caring for our future*, published in 2012 [9]. A number of local areas were selected as *Integration Pioneers* to explore mechanisms for more coordinated working. The provisions for greater integration were laid out in the 2014 Care Act for England, with the subsequent requirement for all local health and social care economies to pool some budgets and develop local plans. This ‘*Better Care Fund*’ was implemented from April 2015 and involved around £5.3 bn of identified funding.

Despite integration being at the centre of the policy debate, this position is supported by a limited evidence base. The modest and tentative literature around this issue has focused primarily on the impact of better coordination between LTC and hospital services, addressing mostly the issue of delayed hospital discharge or avoidable admissions to hospitals, with mixed results [10–14]. We contend that the evidence base for integration between primary care and social care is less researched.

Ideally, the evidence would be developed with comparative trials of more integrated systems compared to current arrangements. A national evaluation of the integration pioneers has been commissioned but is not expected to report until June 2020.^1^ Moreover, such studies face significant challenges in addressing attribution (selection) in non-randomised designs and in having sufficiently comprehensive outcome measures [15, 16]. In this paper, we adopt a more pragmatic approach. The overall aim is to provide results about the scale of interdependence between the use of community-based LTC and primary care (GP) services, where the extent of interdependence is highly indicative of the potential for greater coordination (than the current low level) to improve the efficiency of resource allocation.

## Coordination and pooling of budgets

There are many forms of ‘integration’ policy regarding public services. In this paper, we are largely concerned with ‘coordinated commissioning’ whereby the full range of health and care services are funded from a single, pooled budget and where, potentially, there is an alignment of incentives (e.g. through single accountability mechanisms). Coordination can occur at a system level, e.g. as with the Better Care Fund, or at an individual person level. An example of the latter is the policy of integrated personal commissioning (IPCs) [17]. At a system level, service needs are organised by multi-disciplinary commissioning teams (potentially teams comprising consultants, GPs, nurses and social workers), drawing on a pooled budget. An example would be a coordinated service for people with multi-morbidity, working out of a GP practice using a capitated (pooled) budget.

## Rationale for greater coordination

Two main policy rationales to implement policies that create greater coordination are: (i) those which better internalise preventative (externality) effects between the LTC and health systems, and (ii) those which help to correct prevailing sub-optimal allocation of funding between the systems.

The prevention argument is predicated on there being an interdependence in the way that health and LTC services produce utility (or well-being). We define interdependence as occurring where the use of one service type by an individual changes the marginal effectiveness of the other service for that individual. For example, LTC could reduce the health-related needs of the patient through improved nutrition, hydration, mobility, medicine management and social contact, helping to reduce the risk of falls, infections, depression, etc. [11]. LTC can also help people with social and emotional needs, which can also affect health-related quality of life (as well as care-related quality of life) [18]. Similarly, health care interventions could reduce care needs—e.g. from hip replacements to restore mobility, pain relief for arthritis, improved functioning from mitigation of COPD symptoms, etc.—which reduces the benefits/effectiveness of using LTC. Furthermore, where one service produces assessment and diagnosis information (and shares it), this reduces the need (and so marginal effectiveness) of the other service in producing that information.^2^

Taking a system level perspective, these preventative effects create externalities between sectors. For example, more LTC helps people manage their chronic conditions better, so reducing the need for hospital admissions or GP consultations. Another example is where greater provision of LTC allows more timely transfers of care, reducing the need for as many hospital beds or GP appointment slots. Coordination of decision-making between health and social care could allow better internalisation of these externalities.

A second rationale is that more integrated decision-making can help tackle any sub-optimal allocation of funding between health and social care that has arisen due to a lack of coordination historically. In particular, without coordination, public funding need not be allocated so that the marginal utility of service users that accrues to the last £1 spent on each service area is equal. Similarly, taking an extra-welfarist perspective, the two sectors might not be operating with the same opportunity cost thresholds for equivalent incremental improvements in equivalent QALY benefits. This rationale does not require any technical interdependence (no preventative effects) between service areas, just marginal diminishing utility. However, it is also more of a strategic (public) funding issue, and does not necessarily require coordination at the ‘delivery’ level.

Any comprehensive evaluation of whether greater coordination and pooling of budgets is actually cost-effective is beyond the scope of this paper. Instead, we focus on the necessary condition that predicates the externalities argument made above, namely, that LTC and GP services are interdependent. If service areas were entirely independent (for our target population), then the case for coordination—certainly at the operational, delivery level—is largely undermined.

We can explore this hypothesis empirically by estimating whether the use of LTC by an individual has a causal impact on their utilisation of GP services. Because GP services are free (at the point of use) in England, exogenous shocks that affect the price of LTC services should not lead to changes in demand for GP services from individuals that result from *relative price* effects that work through the budget constraint (because with zero prices for GP services, these decisions are not budget constrained). Rather, such LTC price shocks would (only) work through the GP demand if service utilisation was interdependent in the utility function, that is, where the marginal utility of GP services is affected by the utilisation of LTC (and vice versa). Conversely, finding no causal effect would suggest that there was no utility interdependence, which in turn would mean that preventative effects did not exist.

GP services of course have a cost to the public purse and public commissioners influence the supply (capacity) of GP services, so constraining demand. Moreover, a *fully coordinated* public commissioner (with responsibility for both services and with a unified budget) would react to price shocks. But, if the system is currently separately operated, and this is what we assume as a starting point, then GP service funders would not react to price changes in the LTC system, and vice versa. Potentially, coincidental shocks might show apparent interdependence in the use of GP services and LTC. As a result, and more generally to address causation issues (e.g. omitted variables), we used an instrumental variable approach to assess the effects of LTC on GP service utilisation.

Where preventative/externality effects exist, the implementation of more coordinated commissioning policies should generate greater utility/well-being in the population through the internalisation of these externalities, other things equal (unless by chance services happen to be already utilised at the jointly optimised level). We cannot quantify the net benefits of such a policy reform (without specifying a social welfare function), but we might expect the potential benefit to be proportional to the degree of interdependence. In other words, if LTC and GP services show only negligible or no overlap, policies to coordinate public commissioning decisions regarding these services would have limited impact. Alternatively, if the interdependence between services is significant, the opportunities for such policies to be cost-effective are correspondingly greater.

Potentially, long-term care could be provided by informal carers as well as former service providers. There is an extensive literature on the interplay between formal services (health and care) and informal care [19, 20]. The empirical focus of this paper is on formal care services—specifically those provided in the community. Nonetheless, the conceptual arguments would apply to informally provided LTC in affecting demand for GP services, namely, that shocks to informal care supply would only affect demand for GP services if their impact on utility is interdependent. Policies that affect informal care might be differently configured if a coordinated approach rather than separate decision-making was implemented.

## Aims and structure

Drawing together the above arguments, the paper has two aims. The first is in regard to the hypothesis that LTC and health services are interdependent. In particular, we aim to assess whether the use of community-based LTC services reduces the utilisation of GP services, other things equal. The second aim is to interpret this finding in terms of the case for implementing more coordinated commissioning between GP and LTC services.

## Conceptual framework

### Individual-level decisions

We begin by considering individuals’ demand for doctor consultations. Suppose the utility function of the older person is:

1 $${U_i}={u_i}({x_i},{y_i};{\sigma _i})+{e_i}({x_i};\,{\sigma _i})+{v_i}({m_i}).$$

Here, ${x_i}$ denotes the use of health care—in this case, consultations with a doctor—and ${y_i}$ is the use of long-term care. As noted above, LTC, ${y_i}$, in this case could be provided by informal carers as well as former service providers. A separate treatment of formal and informal care would involve trade-offs between these areas, as well as with (formal) health services. We concentrate on potential substitution between care and health services, assuming that at least part of the utilisation ${y_i}$ comprises formal care services.

Consumption of other goods, services and leisure activities is given by ${m_i}$. We assume that doctor consultations, whilst having a positive impact on health and so utility (through the function ${u_i}$), can also have a negative effect, perhaps through the effort of visiting a doctor, or in terms of the waiting times to see a doctor. This effect, which we call effort for shorthand, is denoted by ${e_i}$ in the utility function. We also assume LTC services have a positive benefit, producing well-being.

A range of risk factors and other need variables act as parameters of Eq. (1), as denoted by ${\sigma _i}$. These would include the severity of the person’s condition, but also other related factors such as health literacy. Finally, utility accrues to other consumption as determined by the function ${v_i}$.

We assume that all elements of the utility function embody diminishing marginal utility, i.e. $u^{\prime}>0,~u^{\prime\prime}<0$ and $v^{\prime}>0,~v^{\prime\prime}<0$. The effort associated with accessing health care produces negative utility, and increasingly so with health care use: $e^{\prime}<0$ and $e^{\prime\prime}<0$.

Individuals have a budget constraint of: ${B_i}={p_i}{y_i}+{m_i}$, where ${p_i}$ is the (generally subsidised) price of LTC paid by client.

In practice, it will be difficult for people to determine optimal service use, but through influence by professionals and from experience, we assume that behaviour approximates the optimal. Solving Eq. (1) gives optimal utilisation as functions of the exogenous variables. For health and LTC services, we have: $x_{i}^{*}=x_{i}^{*}\left( {{p_i},{B_i},{\sigma _i}} \right)$ and $y_{i}^{*}=y_{i}^{*}({p_i},{B_i},{\sigma _i})$, respectively.

Assessing the comparative statics results of an exogenous shock that leads to an increase in the price of LTC services (${p_i}$) using Cramer’s rule, we find that GP service demand is increased, that is, $\frac{{\partial x_{i}^{*}}}{{\partial {p_i}}}>0$, if services are interdependent as substitutes, i.e. if ${u_{xy}}<0$.^3^ Conversely, if services are independent, i.e. ${u_{xy}}=0$, then there is no substitution effect: $\frac{{\partial x_{i}^{*}}}{{\partial {p_i}}}=0$. These results arise because GP service has zero price and so does not enter the budget constraint. Moreover, we assume that there is no binding ‘time’ constraint (since GP consultations and home care visits take a relatively short time). These results underpin our main empirical hypothesis.

### Implications for planning services

Where the effects of LTC and health services are interdependent, then we might expect coordinated decision-making in the public care system to produce better outcomes than separate decision-making between LTC and health care systems for a number of reasons.

The main argument is that interdependence implies externalities and these should be accounted for in resource allocation decisions. We can make the salient points regarding the implications of policies to better integrate or coordinate care by using two representative decision-makers (DMs): for health care (the GP, denoted by *H*) and for LTC (a care manager, or *L*). Decisions are made about the availability of services and the eligibility of patients or clients. Without loss of generality, suppose there are two service users, one with relatively straightforward needs ($i=A$), and one with complex needs ($i=B$). In the former case, externalities effects are smaller than with the latter.

Suppose the objective function for the health DM (*H*) is:

2 $${Z^H}={h^A}\left( {x_{A}^{P}} \right)+{h^B}\left( {x_{B}^{P}} \right),$$

and for the LTC DM (*L*):

3 $${Z^L}={w^A}\left( {y_{A}^{P}} \right)+{w^B}\left( {y_{B}^{P}} \right),$$

where $h$ is the health-related quality of life of the individual, and $w$ is their care-related quality of life, such that $h^{\prime}>0$, $h^{\prime\prime}<0$, $w^{\prime}>0$ and $w^{\prime\prime}<0$. In these functions, ${x^P}$ is the planned level of health care utilisation for the patient/client and ${y^P}$ is LTC utilisation. Through the agency relationship with the patient/client [21, 22], the care professional aims for the client/patient to use planned level of care, reflecting their preferences embodied in ${u_i}$ in Eq. (1). Actual use will differ from planned use in practice, especially if service prices (monetary and otherwise) are subsidised. It suffices that planned supply will be positively correlated with actual utilisation. We assume that if there are interdependencies between ${x_i}$ and ${y_i}$ then we can expect similar interdependencies between the planned levels of $x_{i}^{P}$ and $y_{i}^{P}$ in the DM’s objective function (which follows if the individual’s utility is nested in the DM’s objective function).

A standard externality result is that decisions taken that account for external effects, that is coordinated decisions, will produce optimal resource allocations that are different from those allocations if decisions are taken independently. The coordinated decision-making (denoted *PI*) case is:

4 $$\hbox{max}\!:~Z={h^A}\left( {x_{A}^{{PI}},y_{A}^{{PI}}} \right)+{h^B}\left( {x_{B}^{{PI}},y_{B}^{{PI}}} \right)+{w^A}\left( {x_{A}^{{PI}},y_{A}^{{PI}}} \right)+{w^B}\left( {x_{B}^{{PI}},y_{B}^{{PI}}} \right),$$

$${\text{subject}}~\;{\text{to}}\!:~b={b^H}+{b^L}={c_x}x_{A}^{{PI}}+{c_x}x_{B}^{{PI}}+{c_y}y_{A}^{{PI}}+{c_y}y_{B}^{{PI}}~,$$

where $b$ is the DM’s budget and ${c_x}$ and ${c_y}$ are the unit costs of services. In this case, the costs of GP services are positive for the DM. The usual first-order conditions imply:

5 $$h_{{{x_A}}}^{A}\left( {x_{A}^{{PI*}}} \right)=h_{{{x_B}}}^{B}\left( {x_{B}^{{PI*}}} \right)+w_{{{x_B}}}^{B}\left( {x_{B}^{{PI*}}} \right) - w_{{{x_A}}}^{A}\left( {x_{A}^{{PI*}}} \right),$$

and

6 $$w_{{{y_A}}}^{A}\left( {y_{A}^{{PI*}}} \right)=w_{{{y_B}}}^{B}\left( {y_{B}^{{PI*}}} \right)+h_{{{y_B}}}^{B}\left( {y_{B}^{{PI*}}} \right) - h_{{{y_A}}}^{A}\left( {y_{A}^{{PI*}}} \right).$$

Compared to separate decision-making, coordinated decision-making would (generally) produce a different allocation both between service areas and between the two service users—for example, comparing Eq. (5) with the usual optimal of $h_{{{x_A}}}^{A}\left( {x_{A}^{{PS*}}} \right)=h_{{{x_B}}}^{B}\left( {x_{B}^{{PS*}}} \right)$ where decisions are taken independently; or likewise comparing Eq. (6) with $w_{{{y_A}}}^{A}\left( {y_{A}^{{PS*}}} \right)=w_{{{y_B}}}^{B}\left( {y_{B}^{{PS*}}} \right)$. These results follow from our assumption that $w_{{{x_B}}}^{B} \ne w_{{{x_A}}}^{A},~\forall x$ and $h_{{{y_B}}}^{B} \ne h_{{{y_A}}}^{A},~\forall y$. The interdependence in the individuals’ utility function (i.e. ${u_{xy}} \ne 0$) is the basis for assumption. This is a standard externality problem whereby the internalisation of external benefits leads to a Pareto improvement (subject to transaction costs).

In the above case, a move to coordinated decision-making produces a reallocation of resources because externality effects are internalised. However, coordination could also lead to a reallocation in the case where no externality effects existed because separately determined (global) budgets need not be set optimally, with account being made of the relative marginal benefits of additional public funding for both GP and LTC services (the second rationale as discussed in the introduction).^4^ In this case, we are referring to ‘coordination’ as being at the global or strategic level (in setting global budgets).

From a societal perspective, assuming the public system aimed to maximise both health and well-being (equally weighted in this case), the optimisation problem of the coordinated decision-maker Eq. (4) also defines the socially optimal allocation. Accordingly, the coordinated allocation is preferred to the separately determined allocation, subject to the net benefits outweighing any transaction costs of implementing this new approach.

Current decision rules appear not to account for externalities. If we find evidence of interdependency of services in the individual’s utility function, i.e. if ${u_{xy}} \ne 0$, then we can infer that a more coordinated decision-making process could improve efficiency, as outlined above.

## Empirical specification

Our main empirical hypothesis, $\frac{{\partial x_{i}^{*}}}{{\partial {p_i}}}>0$, is tested using an observational approach with survey data. We cannot directly observe the market price of care services, ${p_i}$, as they affect person $i$. Rather, we exploit the assumptions that person $i$ is a price taker in local market $k~$ (i.e. ${p_{i \in k}}$ or ${p_k}$ for short is also exogenous). The optimal utilisation function for GP services is $x_{i}^{*}=x_{i}^{*}\left( {{p_k},{B_i},{\sigma _i}} \right)$, solving Eq. (1) subject to the budget constraint.

Individuals’ decisions about use of care services will also be positively correlated with exogenous supply factors in local markets, via the change in price that they face. Accordingly, the effects of a change in price due to exogenous shocks is reflected in the change in the amount of care that the person uses following a price change that results from the shock, other things equal. We can also solve for a partial reduced-form function for care services for any given value of GP service use: ${\hat {y}_i}={\hat {y}_i}({p_k},{B_i},{\sigma _i};\,{x_i})$. As individuals are price takers, we can assume $\frac{{\partial {{\hat {y}}_i}\left( {{x_i}} \right)}}{{\partial {p_k}}}<0$, i.e. service demand is inversely related to price, other things equal. As a function of given ${x_i}$ and exogenous variables, we can invert this function for ${p_k}$ and substitute into the optimal GP services function:

7 $$x_{i}^{*}={x_i}({\hat {y}_i},{B_i},{\sigma _i}),$$

where $\frac{{\partial x_{i}^{*}}}{{\partial {{\hat {y}}_i}}}=\frac{{\partial x_{i}^{*}}}{{\partial {p_i}}}/\frac{{\partial {{\hat {y}}_i}}}{{\partial {p_i}}}$. We cannot directly observe ${\hat {y}_i}$, but this variable can be predicted using data on ${y_i}$ and an instrumental variable, ${Z_i}$ (that is not a function of ${x_i}$) in a first-stage reduced form estimation: ${y_i}={y_i}\left( {{\sigma _i},{B_i},{Z_i}} \right)+{\mu _i}$ (i.e. ${\hat {y}_i}={y_i}$ setting ${\mu _i}=0$). Our main hypothesis $\left( {\frac{{\partial x_{i}^{*}}}{{\partial {p_i}}}>0} \right)$ is supported if we find that $\frac{{\partial x_{i}^{*}}}{{\partial {{\hat {y}}_i}}}=\frac{{\partial x_{i}^{*}}}{{\partial {p_i}}}/\frac{{\partial {{\hat {y}}_i}}}{{\partial {p_i}}}<0$ as we assume that $\frac{{\partial {{\hat {y}}_i}}}{{\partial {p_i}}}<0$. In other words, price is negatively correlated with ${\hat {y}_i}$ and positively correlated with ${x_i}$ by hypothesis.

Instrumenting in this way also help address any endogeneity arising from omitted variables in the control factors, ${\sigma _i},{B_i}$. People with high risk/need factors are more likely to use both doctor and LTC services than those with low need levels. Using an IV approach, ${\hat {y}_i}$ should not be correlated with any omitted control factors which instead appear in the error term.

As an instrument, we constructed a ‘spatial lag’ variable: ${\bar {y}_{j \ne i \in {L_j}}}=\mathop \sum \nolimits_{{j \ne i}} {y_{j \in {L_i}}}/{n_{{L_i}}}$, that is, for each person $i$, we calculate the average LTC use by respondents in our data in the person’s local region, ${L_i}$, excluding that person’s own use of services. The idea is that other people’s average use of LTC services in the same local (regional) market will be correlated with person $i$’s use of services due to common supply and local authority policy factors in that market. At the same time, other people’s average regional use of LTC services should not have any direct effect on an individual person $i$’s decision to visit a GP.

The empirical model that accounts for endogeneity is therefore the following:

8 $${V_{it}}=a+{\beta _1}H{H_{it}}+X_{{it}}^{'}{\beta _2}+{\beta _3}{T_i}+{u_i}+{\varepsilon _{it}},$$

9 $$H{H_{it}}={\gamma _0}+X_{{it}}^{'}{\gamma _1}+{\gamma _2}{Z_{it}}+{\gamma _3}{T_i}+{\upsilon _{it}}.$$

${V_{it}}$ is the number of GP visits and $H{H_{it}}$ is the use of home help of individual *i* in year *t*. $X_{{it}}^{'}$ is a vector of individual characteristics that are expected to affect demand such as demographic and needs factors as well as a dummy for living in London to reflect the particular circumstances of the capital, not least the high supply price of services. ${T}_{i}$ is a year dummy, ${u}_{i}$ are individual unobserved effects. ${Z}_{it}$ is the instrumental variable, in our case the average home help use in individual’s $i$ local region, ${L}_{i}$, excluding own utilisation.^5^${\epsilon }_{it}$ and ${\upsilon }_{it}$ are zero-mean error terms.

## Data

### Data and variables

Data for England is taken from the British Household Panel Survey (BHPS), for the years 1991–2009. The BHPS is one of the main longitudinal surveys for studying social issues in Britain. It comprises a nationally representative sample of around 5000 households and 10,000 individuals recruited in 1991 and re-interviewed each year until 2009. If individuals split off from original households to form new households, they are followed and all adult members of these households are also interviewed. Similarly, new members joining sample households become eligible for interview and children are interviewed as they reach the age of 16 years. The questionnaire includes items that span a large number of areas including among others socioeconomic structure, family structure, wealth, consumption, the labour market, health and well-being.

The BHPS has information on both GP and LTC utilisation. In terms of GP utilisation, the survey asks the respondents to report the number of GP consultations they had in the previous year; these are recorded as a categorical variable and respondents can choose between five categories: zero, one to two, three to five, six to ten and ten or more. In the analysis, we both use the variable as a categorical one and also convert it into a ‘pseudo-continuous’ format to estimate the size of the substitution effects using the midpoint of each category for that purpose. As estimation results could be potentially sensitive to replacement values for the open-ended top category, we triangulated with other datasets. (Unpublished) analysis of administrative data on GP consultations by over 75 s in a locality in England suggested that consultation rates of ten or more averaged about 20 consultations per year and we therefore used this value.

With regard to LTC utilisation, BHPS records information about the use of home help. In particular, respondents are asked whether they used home help in the previous year and they respond with a yes or no.

A number of need factors and demographic characteristics are included as control variables in the analysis. These include gender, age, marital status, number of limitations with activities of daily living (ADL),^6^ subjective health status, smoking status, problems with sight, hearing, arm or leg, skin, breathing, stomach, diabetes, anxiety, alcohol and drugs, epilepsy, migraine, heart, blood and others. We also control for a London dummy and year dummies.

Since the oldest in the population are more likely to be the heaviest users of both primary care and home help services, the sample was restricted to people aged 75 years and over. There were a few missing values in the number of GP visits, smoking status and ADL limitations questions. However, when ADL count was the same in the years before and after the year with missing ADL count, this was replaced with the value from the adjacent waves.^7^ The final sample size is 10,177 observations.

### Descriptive statistics

Table 1 shows the distribution of the number of GP visits. On average, respondents had around five GP visits in the previous year. Approximately, 86% of the sample had at least one GP visit in the previous year and 14% reported no visit. Around 13% of the sample used home help in the previous year. Those using home help reported on average a higher number of GP visits (seven visits) compared to those not using home help in the previous year (five visits). Thus, we see that in the raw data there is a positive correlation between GP visits and home help use. This correlation is likely to be a result of endogeneity driven by simultaneity and omitted variables. In the empirical analysis, we control for a number of confounding factors and use an instrumental variables approach to overcome this issue.

**Table 1:** Number of GP consultations in the last year

Categorical	Pseudo-continuous	*N*	Sample (%)
All people 75 +		10,177	
0	0	1397	13.73
1–2	1.5	3123	30.69
3–5	4	2894	28.44
6–10	8	1515	14.89
10 +	20	1248	12.26
More than one		8780	86.28
Mean	5.24		
Variance	36.35		
Skewness	1.65		
Kurtosis	4.54		
People 75 + years using home help		1282	12.60
Mean	7.17		
SD	7.04		
People 75 + years not using home help		8895	87.40
Mean	4.96		
SD	5.82		

BHPS data; years 1991–2009; England only; age 75 years and above

Table 2 presents the sample descriptive statistics. Approximately, 61% of the sample consists of women and the average age is 81 years. 38% of the sample report being married and a 10% are smokers. The majority do not report any ADL limitation (61%). Of the other 39% that reported at least one ADL limitation, most reported only one limitation (12.8% of the sample). Hearing, arm/leg, and heart/blood problems are the most common ones. The majority report an excellent or good subjective health status (53%).

**Table 2:** Descriptive statistics

	*N*	Mean	SD	Min	Max
Service use					
GP visits (categorical)	10,177	2.81	1.21	1	5
GP visits (pseudo-continuous)	10,177	5.24	6.03	0	20
Home help	10,177	0.13	0.33	0	1
Personal characteristics					
Female	10,177	0.61	0.49	0	1
Age	10,177	80.7	4.55	75	100
Married	10,177	0.38	0.49	0	1
Smoker	10,177	0.10	0.30	0	1
Health condition/impairment					
ADL count	10,177	0.82	1.27	0	4
Sight	10,177	0.20	0.40	0	1
Hearing	10,177	0.32	0.47	0	1
Arm/leg/hand	10,177	0.60	0.49	0	1
Heart/blood	10,177	0.45	0.50	0	1
Skin	10,177	0.09	0.29	0	1
Breathing	10,177	0.20	0.40	0	1
Stomach	10,177	0.12	0.33	0	1
Diabetes	10,177	0.09	0.29	0	1
Anxiety/depression	10,177	0.09	0.29	0	1
Alcohol/drugs	10,177	0.00	0.03	0	1
Epilepsy	10,177	0.00	0.06	0	1
Migraine	10,177	0.04	0.21	0	1
Other	10,177	0.07	0.26	0	1
Health over the last 12 months					
Excellent/good	10,177	0.53	0.50	0	1
Fair	10,177	0.31	0.46	0	1
Poor/very poor	10,177	0.16	0.36	0	1
Region					
London	10,177	0.09	0.29	0	1
Instrument					
Average home help use by region	10,177	0.13	0.04	0.06	0.20

BHPS data; years 1991–2009; England only; age 75 years and above

## Results

### Primary findings

Our identification strategy relies primarily on the use of the ‘spatial lag’ as an instrument. We therefore need to test for instrument relevance and validity for our empirical analysis to be robust. Table 3 presents the first-stage results of the IV model. The instrument is a strong predictor of the endogenous variable in the first stage. The coefficient is positive and significant at the 1% level. The underidentification test (Kleibergen-Paap rk LM statistic) rejects the null hypothesis indicating that the model is identified and the excluded instrument is relevant. Furthermore, a weak identification test (Anderson–Rubin Wald test) rejects the null hypothesis that the coefficients of the excluded instruments are jointly equal to zero, suggesting that our instrument is strongly correlated with the endogenous variable. Overall, we are confident that the ‘spatial lag’ variable works well in the first stage. We cannot directly test the validity of the instrument, but following the literature we assess whether the instrument is balanced with respect to the needs-related characteristics of service users [23]. In particular, we split the sample into two groups: those with the instrument above and those below its median value (Table 4). Finding no difference in the mean value of each covariate between groups would provide some reassurance that the instrument is not directly correlated with the dependent variable (in a way that we cannot control for). We calculated the standardised mean difference for each variable between the two groups. Some variables showed a difference of above 0.1 (e.g. ADL count, smoker, good health status), but overall the covariates were reasonably balanced. Accordingly, we had reasonable confidence that the instrument was not systematically correlated with the GP service use dependent variable by some unobserved process (where clearly we were controlling for any direct effect from the included covariates). In “Robustness tests”, we investigate this further by controlling for north, south or midlands dummies as well as a similarly constructed spatial lag variable for the number of doctor visits.

**Table 3:** First-stage results

	Home help
Spatial lag	0.681*** (0.142)
Female	0.019* (0.011)
Age	− 0.025 (0.039)
Age squared	0.0002 (0.0002)
Married	− 0.070*** (0.010)
ADL count	0.040*** (0.013)
ADL count squared	0.004 (0.004)
Health: fair	0.017* (0.009)
Health: poor/very poor	0.080*** (0.017)
Smoker	− 0.030** (0.015)
Sight problem	0.035** (0.014)
Hearing problem	0.003 (0.011)
Arm/leg/hand problem	0.012 (0.008)
Skin problem	0.024 (0.016)
Breathing problem	0.004 (0.013)
Stomach problem	0.024 (0.015)
Diabetes problem	− 0.027* (0.016)
Anxiety/depression problem	0.018 (0.016)
Alcohol/drugs problem	0.014 (0.112)
Epilepsy problem	− 0.041 (0.042)
Migraine problem	0.010 (0.023)
Other problem	0.004 (0.016)
Heart blood problem	0.013 (0.009)
London region	0.005 (0.019)
Constant	0.477 (1.573)
Year dummies	Yes
K-P rk LM statistic [chi-sq(1)] (under-id)	22.09***
Anderson-Rubin Wald test [chi-sq(1)] (weak-id)	8.47***
Observations	10,177

Pooled 2sls model. ***p < 0.01, **p < 0.05, *p < 0.1. Statistics robust to heteroskedasticity and clustering on individual level. Reference category: excellent/good health

**Table 4:** Individual-level characteristics by regional home help utilisation

	All		IV below the median		IV above the median		Standardised mean diff.
	Mean	SD	Mean	SD	Mean	SD	
Personal characteristics							
Female	0.61	0.49	0.59	0.49	0.62	0.48	− 0.06
Age	80.70	4.55	80.71	4.60	80.70	4.49	0.00
Married	0.38	0.49	0.40	0.49	0.36	0.48	0.09
Smoker	0.10	0.30	0.08	0.27	0.12	0.33	− 0.16
Health conditions/impairments							
ADL count	0.82	1.27	0.74	1.23	0.92	1.31	− 0.14
Sight	0.20	0.40	0.20	0.40	0.19	0.39	0.03
Hearing	0.32	0.47	0.31	0.46	0.33	0.47	− 0.04
Arm/leg/hand	0.60	0.49	0.58	0.49	0.62	0.48	− 0.08
Skin	0.09	0.29	0.09	0.29	0.09	0.28	0.01
Breathing	0.20	0.40	0.18	0.38	0.22	0.41	− 0.11
Stomach	0.12	0.33	0.11	0.31	0.14	0.35	− 0.09
Diabetes	0.09	0.29	0.10	0.30	0.08	0.27	0.05
Anxiety/depression	0.09	0.29	0.09	0.28	0.10	0.30	− 0.05
Alcohol/drugs	0.00	0.03	0.00	0.03	0.00	0.04	− 0.01
Epilepsy	0.00	0.06	0.00	0.05	0.00	0.06	− 0.02
Migraine	0.04	0.21	0.05	0.21	0.04	0.20	0.02
Other	0.07	0.26	0.07	0.26	0.07	0.25	0.03
Heart blood	0.45	0.50	0.47	0.50	0.43	0.49	0.08
Subjective health status							
Excellent/good	0.53	0.50	0.56	0.50	0.50	0.50	0.12
Fair	0.31	0.46	0.30	0.46	0.33	0.47	− 0.06
Poor/very poor	0.16	0.36	0.14	0.35	0.17	0.38	− 0.09
*N*	10,177		5646		4531		

Balance tests compare for systematic differences in the control variables between the ‘treated’ and ‘control’ groups (here, above and below the IV median)

With regard to the main estimation, four estimators were used: a pooled 2sls model, a random effects ordered probit model, a random effects model (both with manually instrumented home help use) and a random effects IV model. Standard errors were clustered at the individual level to account for repeated observations, and bootstrapped in the random effects IV model. Random effects are at the individual level. The main model of reference is the random effects ordered probit so as not to impose additional assumptions on the distribution of the dependent variable. The other models are presented for comparison as well as a way to test the sensitivity of the results to the estimator used. While the use of the random effects estimators requires the additional assumption that individual effects are uncorrelated with the explanatory variables compared to a fixed effects model, a Hausman test (13.20) for the choice between the two models failed to reject the null hypothesis providing support for the use of a random effects estimator which is more efficient.

**Fig. 1:**
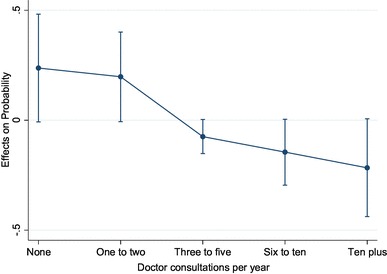
Average marginal effects of home help (predicted) with 95% CIs

Table 5 presents the primary estimation results. There was evidence of endogeneity of the home help variable as expected. The results from all four models provided support for our main hypothesis. Home help use has a statistically significant and negative effect on the number of GP consultations (contrasting with the raw correlation of these variables). Table 6 and Fig. 1 present the estimated marginal effects of home help use on the probability of being in each of the GP visits categories (using the random effects ordered probit model results). We see that using home help significantly increases the probability of being in the low-frequency categories (0 or 1–2 consultations) and significantly lowers the probability of being in the high-frequency categories (3–5, 6–10, 10 + consultations). Applying midpoint notional values (as listed in the table) the average marginal effect of having used home help was on average a 5.5 reduction in GP visits in the year. The models using the pseudo-continuous variable tended to produce slightly higher marginal effects (of − 6.6 to − 10.7).

**Table 5:** Effect of using home help on the number of GP consultations

	Pooled 2sls (1)	RE ordered probit (2)	Random effects (3)	IV random effects (4)
Home help	− 9.359** (3.759)	− 1.563* (0.823)	− 6.591** (3.263)	− 10.670* (6.137)
Female	0.241 (0.235)	0.085 (0.052)	0.236 (0.200)	0.502* (0.301)
Age	− 0.433 (0.589)	− 0.018 (0.106)	− 0.144 (0.431)	− 0.432 (0.621)
Age squared	0.003 (0.004)	0.0001 (0.001)	0.001 (0.003)	0.003 (0.004)
Married	− 0.604* (0.341)	− 0.041 (0.073)	− 0.363 (0.287)	− 0.370 (0.269)
ADL count	1.281*** (0.296)	0.227*** (0.053)	1.030*** (0.238)	1.108*** (0.236)
ADL count squared	− 0.133* (0.076)	− 0.024* (0.012)	− 0.081 (0.058)	− 0.099** (0.045)
Health: fair	1.936*** (0.192)	0.545*** (0.036)	1.901*** (0.148)	1.850*** (0.168)
Health: poor/very poor	5.286*** (0.447)	1.097*** (0.085)	4.661*** (0.367)	4.766*** (0.621)
Smoker	− 1.267*** (0.331)	− 0.331*** (0.073)	− 0.939*** (0.272)	− 0.992*** (0.291)
Sight problem	0.449 (0.278)	0.095* (0.050)	0.275 (0.208)	0.198 (0.158)
Hearing problem	0.093 (0.198)	0.047 (0.035)	− 0.005 (0.149)	0.051 (0.174)
Arm/leg/hand problem	0.570*** (0.169)	0.191*** (0.034)	0.426*** (0.131)	0.480*** (0.176)
Skin problem	0.828*** (0.319)	0.155*** (0.054)	0.641*** (0.238)	0.621** (0.308)
Breathing problem	1.230*** (0.256)	0.221*** (0.040)	0.759*** (0.178)	0.642*** (0.185)
Stomach problem	0.938*** (0.307)	0.206*** (0.049)	0.531** (0.220)	0.351 (0.299)
Diabetes problem	0.659* (0.353)	0.189*** (0.073)	0.610* (0.339)	0.551 (0.349)
Anxiety/depression problem	1.081*** (0.354)	0.252*** (0.053)	0.940*** (0.258)	0.945*** (0.299)
Alcohol/drugs problem	1.061 (1.620)	− 0.204 (0.515)	− 1.271 (2.294)	− 0.672 (3.355)
Epilepsy problem	− 0.364 (1.135)	0.150 (0.253)	0.484 (1.288)	1.048 (1.150)
Migraine problem	− 0.169 (0.456)	0.016 (0.073)	0.005 (0.326)	0.155 (0.388)
Other problem	0.981*** (0.321)	0.162*** (0.054)	0.621** (0.246)	0.634** (0.268)
Heart blood problem	1.868*** (0.184)	0.386*** (0.033)	1.290*** (0.140)	1.138*** (0.158)
London region	− 0.149 (0.369)	− 0.074 (0.079)	− 0.221 (0.306)	− 0.097 (0.282)
Constant	17.040 (23.900)		8.010 (17.650)	18.050 (23.830)
Cut points				
1		− 1.479 (4.353)		
2		− 0.118 (4.354)		
3		0.977 (4.355)		
4		1.795 (4.356)		
Year dummies	Yes	Yes	Yes	Yes
Observations	10,177	10,177	10,177	10,177
Under-id (K-P rk LM statistic) [Chi-sq(1)]	22.09***			
Weak-id (K-P rk Wald *F* statistic/ *F*-stat)	22.98***	86.18***	86.18***	86.18***
Endogeneity (Durbin–Wu–Hausman statistic) [Chi-sq(1)]	9.19***	5.31*	4.66*	4.66*

***p < 0.01, **p < 0.05, *p < 0.1. Clustered standard errors at the individual level (models 1, 2, 3) and bootstrapped standard errors (model 4) in parentheses. Reference category: excellent/good health. Excluded instrument: average regional home help use excluding own utilisation. Manually instrumented home help use (models 2 and 3)

**Table 6:** Conditional marginal effect of home help on the probability of each outcome (random effects ordered probit)

GP visits	Marginal effect	95% Confidence interval		Notional amount (midpoint)
Outcome 1: none	0.24	− 0.01	0.48	0
Outcome 2: one to two	0.20	− 0.01	0.40	1.5
Outcome 3: three to five	− 0.07	− 0.15	0.00	4
Outcome 4: six to ten	− 0.15	− 0.30	0.00	8
Outcome 5: more than ten	− 0.22	− 0.44	0.01	20

As regards the other control variables, health and needs variables were strong predictors of the number of GP visits. A higher ADL limitations count was statistically significant and led to a higher number of GP visits. Similarly, having problems with arm or leg, skin, breathing, diabetes, anxiety and heart or blood had a positive and statistically significant effect on the number of GP visits. Subjective health status was also significant with those reporting fair and poor health having a higher number of visits compared to those reporting good health. Being a smoker was associated with a smaller number of GP visits, which was somewhat contrary to expectations but could reflect risk-taking behaviour as opposed to a needs factor.

We might expect that the preventative effects of home help use are stronger for people with higher condition severity ${\sigma _i}$ and consequently higher need for long-term care compared to people with low risk. Existing evidence suggests that LTC services have a greater effect on well-being for people with high levels of need rather than low levels [11, 24]. This result stems from people having a greater capacity to benefit from services if they have greater levels of impairment [25, 26].

The size of the substitution effect can also be expressed in cost terms (i.e. $\frac{{\partial x_{i}^{*}}}{{\partial {{\hat {y}}_i}}}\frac{{{{\bar {c}}_x}}}{{{{\bar {c}}_y}}}$). Assuming a unit cost of £46 per GP consultation (${\bar {c}_x}$) and £150 per week or £7,800 per year for home help $({\bar {c}_y})$, using national unit cost figures [27], the results show that every extra £1 spent on home help services will generate a reduction in demand for GP services equivalent to approximately £0.03 per annum on average (using the ordered probit results).

### Robustness tests

As robustness tests, we controlled for additional covariates to exclude possible correlation of the instrument with demand, and estimated different instrument specifications.

All the results are reported in Table 7. First, we estimated a model with the spatial lag square as an additional instrument (Table 7; column 1). The Hansen *J* statistic showed no problem with overidentification restrictions. Secondly, we estimated a model with further income-related proxies, specifically a dummy for home ownership and a dummy for whether the respondents receive attendance allowance (Table 7; column 2). The third variant added controls for regional differences to the main model, specifically dummies for north, midlands and south of England (Table 7; column 3) and a spatially lagged number of GP visits (Table 7; column 4). In these specifications, the results regarding the effect of home help do not substantially change in terms of sign or magnitude. In addition, the effect of home ownership, attendance allowance receipt or spatially lagged GP visits was not statistically significant, while only the south dummy was significant compared to the north dummy.^8^ Furthermore, since subjective health status can be seen as an outcome itself, we estimated a model excluding it from the controls list (Table 7; column 5). Again, the effect of home help did not substantially change while the diagnostic tests gave the expected results. Lastly, we test for whether the use of other services can affect the relationship under study. We do not have data on the use of all other possible services, but from BHPS we control for whether individuals had a district nurse or health visitor visit in the last year. We see that although the effect of seeing a district nurse has a positive significant effect on the number of GP visits, the impact of using home help does not change in terms of size, sign or significance.

**Table 7:** Effect of home help use on GP consultations, robustness tests

	1	2	3	4	5	6
Home help	− 9.410** (3.740)	− 9.153** (4.003)	− 8.444** (4.087)	− 9.104** (4.026)	− 8.639** (3.761)	− 9.868** (3.939)
GP visits (spatial lag)	–	–	–	0.055 (0.288)	–	–
District nurse/ health visitor	–	–	–	–	–	1.490*** (0.451)
Year dummies	Yes	Yes	Yes	Yes	Yes	Yes
Observations	10,177	9,987	10,177	10,177	10,183	10,177
Weak-id (K-P rk Wald *F* statistic)	43.72***	20.27***	21.23***	19.51***	23.31***	21.77***
Endogeneity (Durbin–Wu–Hausman statistic) [Chi-sq(1)]	10.07***	8.02***	5.82**	7.91***	8.19***	9.38***
Overidentification test (Hansen *J* statistic) [Chi-sq(1)]	0.43					

Model 1: Excluded instruments: spatial lag (regional average home help use excluding own use), spatial lag squared; other controls as in *X*

Model 2: Excluded instrument: spatial lag; other controls as in *X* and house ownership dummy and attendance allowance dummy

Model 3: Excluded instrument: spatial lag; other controls as in *X* and north/midlands/south dummies with north as the reference category

Model 4: Excluded instrument: spatial lag; other controls as in *X* and ‘spatial lag’ number of doctor visits

Model 5: Excluded instrument: spatial lag; other controls as in *X*, except for subjective health status dummies

Model 6: Excluded instrument: spatial lag; other controls as in *X* and use of district nurse or health visitor

***p < 0.01, **p < 0.05, *p < 0.1; clustered standard errors at the individual level in parentheses; pooled 2sls estimator

## Discussion

There is considerable policy interest in the better integration of long-term care with primary and secondary care, especially in England where long-term care is mostly the responsibility of local government rather than the National Health Service. In this paper, we developed a conceptual framework with a number of theoretical hypotheses which were investigated using data from the BHPS (1991–2009).

We found that (exogenously driven) changes in community-based LTC service (home care) utilisation lead to negatively related changes in the demand for GP consultations. Our main estimates indicated that using home care results in approximately five fewer doctor (GP) consultations in a year.

This substitution effect corresponds to a £0.03 for an extra £1 spent on long-term care, which is relatively small since the cost of long-term care is incurred throughout the year and is high. Nonetheless, this effect estimate will likely be a lower bound of the overall substitution effect because it only accounts for the direct effect of home care on GP service use. GP consultations often result in referrals, prescription costs, etc., so we might assume that where a person has fewer GP consultations, this will be associated with fewer additional health costs of this nature.

Moreover, the results do not include any further benefits from externality effects created by greater coordination, e.g. from sharing of information through joint assessment, reduction in duplicated activity and so on [4].

These findings provide support for policies that seek to increase coordination between health and LTC systems, particularly with respect to primary care.Greater coordination in this case should improve efficiency through a more integrated resource allocation mechanism because, given the current low baseline of integrated decision-making, the spillover effects of decisions in one service area on the other are not factored into those decisions. The size of the substitution effect gives us a sense of the scale of the efficiency improvement: larger effects (in absolute terms) suggest more scope of efficiency improvement. However, we cannot determine the size of any improvement in social welfare or how coordination *should* change resource allocation between the two sectors without (i) results regarding the main effects of services on outcomes and costs (i.e. of ${u}_{x}$ and ${u}_{y}$ and of ${c}_{x}$ and ${c}_{y}$) and (ii) a specification of the social welfare function. We would need more information on the marginal benefit per £1 (or cost-effectiveness) being produced by health and LTC services, and on the transaction costs associated with implementing a more coordinated decision-making system. Furthermore, we would need to know whether, and how, a ‘coordinated’ system would work to achieve, or move towards, the optimal solution. Nonetheless, finding that substitution effects exist, as we do, establishes the case for an exploration of the net benefits of a more coordinated system.

Information on the incremental cost-effectiveness of health and care services is being estimated and collected by public agencies—such as the National Institute of Health and Care Excellence (NICE) in England—but the available evidence base is limited. A number of relevant care-related quality of life measures are available, such as EQ-5D [28] and ASCOT [26] and a recent paper by Stevens et al. [29] has established their relative value.

There were a number of empirical challenges in our analysis, which we should note. To begin with, there was the categorical nature of the dependent variable. Ordered probit as well as linear models (with the use of a pseudo-continuous variable for doctor visits) were used to establish the significance of the effect. The results were consistent with the main substitution hypothesis across this range of models indicating a statistically significant substitution effect between LTC use and primary care visits of similar magnitude.

A second challenge was the potential endogeneity in the LTC use and primary care relationship arising from unobserved omitted variables and reverse causality. To address this issue we used an instrumental variables approach exploiting the spatial structure of the data. Overall, the instrument worked well, with diagnostic and specification tests indicating that it is relevant and valid. However, given that the instrumental variable estimation is sensitive to assumptions, and the exclusion restriction assumption is not directly testable, we still need to be somewhat cautious with the results.

Also, in using a community-based survey, we were unable to assess the impact of residential care on doctor consultation rates. Potentially, the availability of care home services might influence the relationship between community-based social care and doctor consultation rates, which should be controlled for in the analysis. However, given the much greater prevalence of community-based rather than residential social care we do not consider this to be a significant limitation.

In conclusion, this paper has contributed evidence of significant inter-relationships between primary care health services and LTC services for older people, albeit to a modest degree. Historically, there has been little account of these interdependencies and negligible coordination between the two sectors. This analysis provides groundwork for policies that aim to create greater coordination.

## Electronic supplementary material

Below is the link to the electronic supplementary material.

ESM1 (DOCX 41 kb)

## Footnotes

1 http://www.piru.ac.uk/assets/files/Integrated%20Care%20Pioneers%20Evaluation%20%202015-2020-%20one%20page%20summary%20PIRU%2015%20Oct%2015.pdf.

2 Potentially, there could be a countervailing effect whereby diagnostic/assessment information produced by one sector could help better target services in the other sector, given a positive cross-sector effect on marginal effectiveness. In any case, however, these are examples of interdependent effects.

3 This result is established in the electronic supplementary material 1.

4 See electronic supplementary material 2 for more details.

5 Our data records as geographic information the standard regions distinguishing former Metropolitan Counties and Inner and Outer London. Therefore, the local regions at which the instrument is constructed are the following: rest of South East, South West, East Anglia, East Midlands, West Midlands Conurbation, rest of West Midlands, Greater Manchester, Merseyside, rest of North West, South Yorkshire, West Yorkshire, rest of Yorks and Humberside, Tyne and Wear, rest of North. Inner and Outer London are collapsed into one category.

6 ADLs consist of doing housework, climbing stairs, getting dressed, walking for more than 10 min or other.

7 The results did not change when we did not account for the missing values in this way.

8 Results are available upon request.
