# Supplementary material for: The impact of long-term care on primary care doctor consultations for people over 75 years
Source: Eur J Health Econ. 2018 Sep 5;20(3):375–87. doi: 10.1007/s10198-018-0999-6 (PMC6438947; doi:10.1007/s10198-018-0999-6)
Supplement: Supplementary file 1 — ESM1 (DOCX 41 kb) [file 10198_2018_999_MOESM1_ESM.docx]

**Electronic supplementary material 1**

Regarding the utility function Eq. (1) and the budget constraint, the Lagrangian is:

$L_{i}=u_{i}\left( x_{i},y_{i};\sigma_{i} \right)+e_{i}\left( x_{i};\sigma_{i} \right)+v_{i}\left( m_{i} \right)+\lambda\left( B_{i}-m_{i}-p_{i}y_{i} \right)$ (8)

The first order conditions are:

$L_{x}=u_{x}\left( x_{i},y_{i};\sigma_{i} \right)+e_{x}\left( x_{i};\sigma_{i} \right)=0$ (9)

$L_{y}=u_{y}\left( x_{i},y_{i};\sigma_{i} \right)-\lambda p_{i}=0$ (10)

$L_{m}=v_{m}\left( m_{i} \right)-\lambda=0$ (11)

$L_{\lambda}=B_{i}-m_{i}-p_{i}y_{i}=0$ (12)

Taking the total differentials of the FOCs with respect to changes in price gives:

$0d\lambda+0dm^{*}+\left( u_{xx}+e_{xx} \right)dx^{*}+u_{xy}dy^{*}=0dp$ (13)

$-pd\lambda+0dm^{*}+u_{yx}dx^{*}+u_{yy}dy^{*}=\lambda dp$ (14)

$-1d\lambda+v_{mm}dm^{*}+0dx^{*}+0=0dp$ (15)

$0d\lambda-1dm^{*}+0dx^{*}-pdy^{*}=ydp$ (16)

These four conditions can be written in matrix form:

$\left[ \begin{matrix} 0 & 0 & \left( u_{xx}+e_{xx} \right) & u_{xy} \\ -p & 0 & u_{yx} & u_{yy} \\ -1 & v_{mm} & 0 & 0 \\ 0 & -1 & 0 & -p \end{matrix} \right] \left( \begin{matrix} {d\lambda^{*}}/{dp} \\ {dm^{*}}/{dp} \\ {dx^{*}}/{dp} \\ {dy^{*}}/{dp} \end{matrix} \right)=\left( \begin{matrix} 0 \\ \lambda\\ 0 \\ y \end{matrix} \right)$ (17)

We can solve for the impact of a change in the price of LTC on the optimal use of GP services using Cramer’s rule:

$\frac{\partial x^{*}}{\partial p}=\frac{1}{\left| \bar{H} \right|}\left[ \begin{matrix} 0 & 0 & 0 & u_{xy} \\ -p & 0 & \lambda& u_{yy} \\ -1 & v_{mm} & 0 & 0 \\ 0 & -1 & y & -p \end{matrix} \right]$ (18)

where $\bar{H}$ is the bordered Hessian matrix, whose determinant is assumed to be positive to ensure that utility is maximised subject to the constraint in Eq. (8). In turn,

$\frac{\partial x^{*}}{\partial p}=\frac{-u_{xy}}{\left| \bar{H} \right|}\left| \begin{matrix} -p & 0 & \lambda\\ -1 & v_{mm} & 0 \\ 0 & -1 & y \end{matrix} \right|=\frac{-u_{xy}}{\left| \bar{H} \right|}\left( -p\left| \begin{matrix} v_{mm} & 0 \\ -1 & y \end{matrix} \right|+\lambda\left| \begin{matrix} -1 & v_{mm} \\ 0 & -1 \end{matrix} \right| \right)==\frac{u_{xy}}{\left| \bar{H} \right|}\left( pv_{mm}y-\lambda\right)>0$ (19)

The hypothesis that $\frac{\partial x^{*}}{\partial p}>0$ follows from the assumption that $u_{xy}<0$ (and also that $v_{mm}\leq0$). In other words, an increase in the price of LTC will reduce LTC utilisation and in turn increase the use of GP services. Finding $\frac{\partial x^{*}}{\partial p}>0$ in this case is sufficient for us to conclude that $u_{xy}<0$, which is a necessary condition. This result occurs because GP services are free at the point of use and so has no budgetary implication. Where services are independent i.e. $u_{xy}=0$, then from Eq. (19), $\frac{\partial x^{*}}{\partial p}=0$. Alternatively, if people did face a price for GP services, then $\frac{\partial x^{*}}{\partial p}>0$ could arise when $u_{xy}=0$.

**Electronic supplementary material 2**

For illustration, consider the case where the objective functions Eq. (2) and (3) are additive in logs, the budget constraints are as described above and there are now $n$ people in the population:

$Z^{H}=h^{A}log\left( x_{A}^{P} \right)+h^{B}log\left( x_{B}^{P} \right)+\ldots=h^{i}log\left( x_{i}^{P} \right)$ (20)

and

$Z^{L}=w^{A}log\left( y_{A}^{P} \right)+w^{B}log\left( y_{B}^{P} \right)+\ldots=w^{i}log\left( y_{i}^{P} \right)$ (21)

With separate decision making, the first order conditions imply:

$\frac{h^{i}}{x_{i}^{PS}}=\frac{h^{j}}{x_{j}^{PS}}$ (22)

The budget constraint means:

$x_{i}^{PS}=b^{H}-c_{x}\sum_{j\neq i}^{n-1} x_{j}^{PS}=b^{H}-c_{x}\sum_{j\neq i}^{n-1} \frac{h^{j}}{h^{i}}x_{i}^{PS}$ (23)

$x_{i}^{PS}=\frac{b^{H}}{\left( 1+c_{x}\sum_{j\neq i}^{n-1} \frac{h^{j}}{h^{i}} \right)}=\frac{b^{H}}{\left( \frac{c_{x}\sum_{j=1}^{n} h^{j}}{h^{i}} \right)}$ (24)

With coordinated decision-making (i.e. $Z=Z^{H}+Z^{L}$) and no externalities, two of the first order conditions are:

$x_{j}^{PI}=\frac{h^{j}}{h^{i}}x_{i}^{PI}, \forall j\neq i$ (25)

$y_{k}^{PI}=\frac{c_{x}}{c_{y}}\frac{w^{k}}{h^{i}}x_{i}^{PI}, \forall k\neq i$ (26)

So, re-arranging the pooled budget constraint:

$x_{i}^{PI}=b-c_{x}\sum_{j\neq i}^{n-1} x_{j}^{PI}-c_{y}\sum_{k=1}^{n} y_{k}^{PI}$ (27)

$x_{i}^{PI}=b-c_{x}\sum_{j\neq i}^{n-1} \frac{h^{j}}{h^{i}}x_{i}^{PI}-c_{y}\sum_{k=1}^{n} \frac{c_{x}}{c_{y}}\frac{w^{k}}{h^{i}}x_{i}^{PI}$ (28)

$x_{i}^{PI}=\frac{b}{\left( 1+c_{x}\sum_{j\neq i}^{n-1} \frac{h^{j}}{h^{i}}+c_{x}\sum_{k=1}^{n} \frac{w^{k}}{h^{i}} \right)}=\frac{b}{\left( \frac{c_{x}\sum_{j=1}^{n} h^{j}+c_{x}\sum_{k=1}^{n} w^{k}}{h^{i}} \right)}$ (29)

We can assess the conditions whereby separate and coordinated decision-making would give the same levels of service use between the sectors, and then they would differ. In particular, we can calculate the budget allocation required for $x_{i}^{PI}=x_{i}^{PS}$ using Eq. (24) and (29):

$\frac{b}{\left( \frac{c_{x}\sum_{j=1}^{n} h^{j}+c_{x}\sum_{k=1}^{n} w^{k}}{h^{i}} \right)}=\frac{b^{H}}{\left( \frac{c_{x}\sum_{j=1}^{n} h^{j}}{h^{i}} \right)}$ (30)

or

$b^{H}=\frac{\sum_{j=1}^{n} h^{j}}{\sum_{j=1}^{n} h^{j}+\sum_{k=1}^{n} w^{k}}b=\frac{\bar{h}}{\bar{h}+\bar{w}}B$ (31)

In this example, if the budgets in each sector were set arbitrarily in the ratio specified by Eq. (31) then separate and coordinated decision-making would give the same service allocation (other things equal). However, this distribution of budgets is a special case and would have to be purposefully specified through coordination at the global budget-setting level. Without such coordination, only by chance would independently set, separate budget corresponds to this ratio.
